# Supplementary material for: Multisensory interactions on auditory and somatosensory information in expert pianists
Source: Sci Rep. 2022 Jul 22;12:12503. doi: 10.1038/s41598-022-16618-0 (PMC9307509; doi:10.1038/s41598-022-16618-0)
Supplement: Supplementary file 1 — Supplementary Information. [file 41598_2022_16618_MOESM1_ESM.docx]

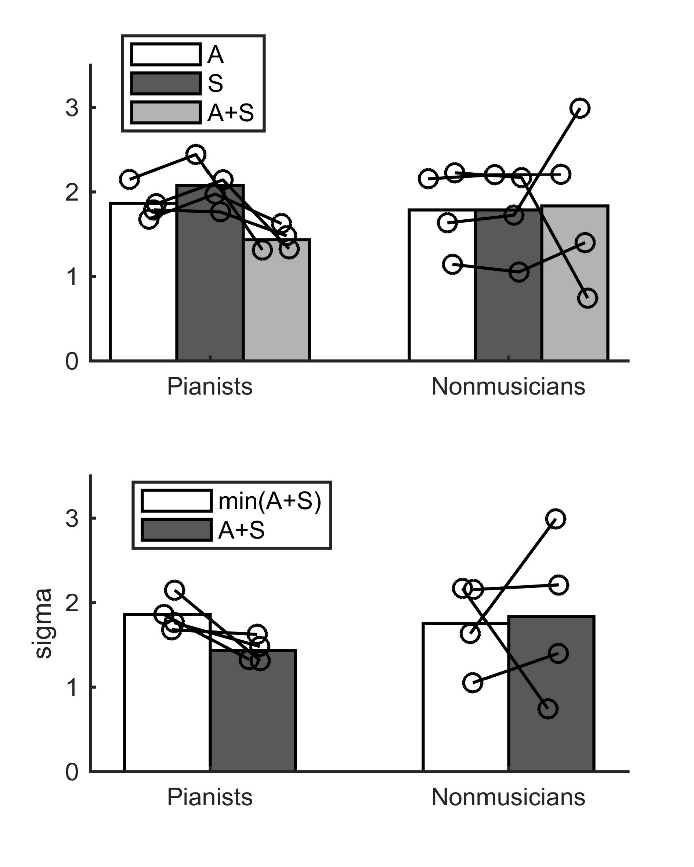


Supplementary Figure 1. Results obtained from the pilot experiment. Upper panel shows the group mean of the sigma value obtained from A, S, and A+S conditions in the pianists and nonmusicians. Lower panel shows the group mean of the sigma value obrained from the A+S condition and the minimum value between the A and S conditions. Each circle represents individual data.


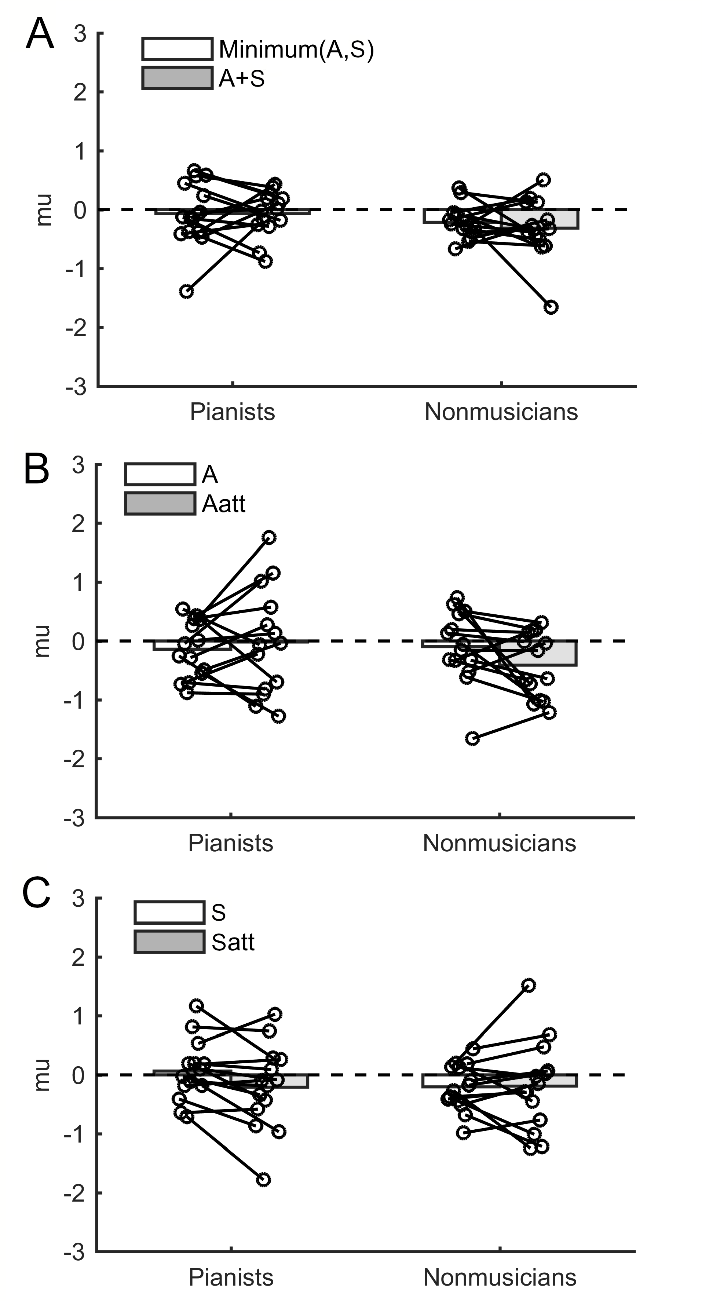


Supplementary Figure 2. Group means of the mu value obtained from each condition in the pianists and nonmusicians. A: The multimodal integration (A+S) vs the minimum sigma value of those obtained from the A and S conditions (minimum(A,S)). B: The unimodal auditory (A) vs auditory selective attention (Aatt) conditions. C: The unimodal somatosensory (S) vs somatosensory selective attention (Satt) conditions. Each circle represents individual data.


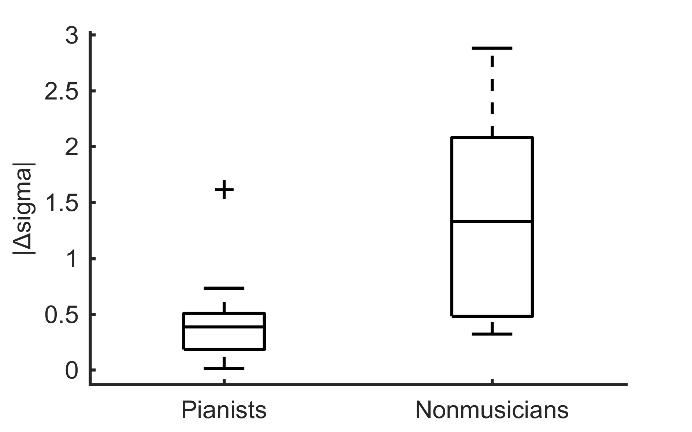


Supplementary figure 3. Box plot of the absolute difference in the sigma value between the A and S conditions. Box consists of the first and third quartiles, and the band inside the box is the second quartile. The lowest end of whisker indicates within 1.5 IQR of the lower quartile, and the highest end of whisker indicates within 1.5 IQR ofthe upper quartile. Any data not included between thewhiskers are plotted as an outlier (plus marks).

| Supplementary table 1. Bayesian Repeated Measures ANOVA for the mu value in A+S condition | | | | | | | | | | | |
| --- | --- | --- | --- | --- | --- | --- | --- | --- | --- | --- | --- |
| **Models** | | **P(M)** | | **P(M\|data)** | | **BF _M_** | | **BF _10_** | | **error %** | |
| Null model (incl. subject) |  | 0.20 |  | 0.37 |  | 2.38 |  | 1.00 |  |  |  |
| Group |  | 0.20 |  | 0.30 |  | 1.69 |  | 0.80 |  | 4.66 |  |
| Condition + Group + Condition  ✻  Group |  | 0.20 |  | 0.13 |  | 0.58 |  | 0.34 |  | 72.04 |  |
| Condition |  | 0.20 |  | 0.12 |  | 0.53 |  | 0.31 |  | 1.69 |  |
| Condition + Group |  | 0.20 |  | 0.09 |  | 0.38 |  | 0.23 |  | 1.11 |  |
|  | | | | | | | | | | | |
| *Note.*  All models include subject | | | | | | | | | | | |

| Supplementary table 2. Bayesian Repeated Measures ANOVA for the mu value in auditory selective attention | | | | | | | | | | | |
| --- | --- | --- | --- | --- | --- | --- | --- | --- | --- | --- | --- |
| **Models** | | **P(M)** | | **P(M\|data)** | | **BF _M_** | | **BF _10_** | | **error %** | |
| Null model (incl. subject) |  | 0.20 |  | 0.47 |  | 3.61 |  | 1.00 |  |  |  |
| Group |  | 0.20 |  | 0.22 |  | 1.16 |  | 0.47 |  | 1.41 |  |
| Condition |  | 0.20 |  | 0.16 |  | 0.73 |  | 0.33 |  | 1.50 |  |
| Condition + Group |  | 0.20 |  | 0.07 |  | 0.32 |  | 0.16 |  | 1.26 |  |
| Condition + Group + Condition  ✻  Group |  | 0.20 |  | 0.07 |  | 0.31 |  | 0.15 |  | 1.64 |  |
|  | | | | | | | | | | | |
| *Note.*  All models include subject | | | | | | | | | | | |

| Supplementary table 3. Bayesian Repeated Measures ANOVA for the mu value in somatosensory selective attention | | | | | | | | | | | |
| --- | --- | --- | --- | --- | --- | --- | --- | --- | --- | --- | --- |
| **Models** | | **P(M)** | | **P(M\|data)** | | **BF _M_** | | **BF _10_** | | **error %** | |
| Null model (incl. subject) |  | 0.20 |  | 0.40 |  | 2.62 |  | 1.00 |  |  |  |
| Condition |  | 0.20 |  | 0.21 |  | 1.09 |  | 0.54 |  | 1.53 |  |
| Group |  | 0.20 |  | 0.20 |  | 0.99 |  | 0.50 |  | 0.93 |  |
| Condition + Group |  | 0.20 |  | 0.11 |  | 0.48 |  | 0.27 |  | 1.21 |  |
| Condition + Group + Condition  ✻  Group |  | 0.20 |  | 0.09 |  | 0.37 |  | 0.22 |  | 2.10 |  |
|  | | | | | | | | | | | |
| *Note.*  All models include subject | | | | | | | | | | | |
